# Acute myocardial infarction hospital admissions and deaths in England: a national follow-back and follow-forward record-linkage study

**DOI:** 10.1016/S2468-2667(17)30032-4

**Published:** 2017-03-01

**Authors:** Perviz Asaria, Paul Elliott, Margaret Douglass, Ziad Obermeyer, Michael Soljak, Azeem Majeed, Majid Ezzati

**Affiliations:** a Department of Epidemiology and Biostatistics, School of Public Health, Imperial College London, London, UK; b UK Small Area Health Statistics Unit, MRC-PHE Centre for Environment and Health, Imperial College London, London, UK; c Department of Primary Care and Public Health, School of Public Health, Imperial College London, London, UK; d Imperial College Healthcare NHS Trust, London, UK; e Department of Emergency Medicine and Health Care Policy, Harvard Medical School, Harvard University, Boston, MA, USA; f Department of Emergency Medicine, Brigham and Women's Hospital, Boston, MA, USA

## Abstract

**Background:**

Little information is available on how primary and comorbid acute myocardial infarction contribute to the mortality burden of acute myocardial infarction, the share of these deaths that occur during or after a hospital admission, and the reasons for hospital admission of those who died from acute myocardial infarction. Our aim was to fill in these gaps in the knowledge about deaths and hospital admissions due to acute myocardial infarction.

**Methods:**

We used individually linked national hospital admission and mortality data for England from 2006 to 2010 to identify all primary and comorbid diagnoses of acute myocardial infarction during hospital stay and their associated fatality rates (during or within 28 days of being in hospital). Data were obtained from the UK Small Area Health Statistics Unit and supplied by the Health and Social Care Information Centre (now NHS Digital) and the Office of National Statistics. We calculated event rates (reported as per 100 000 population for relevant age and sex groups) and case-fatality rate for primary acute myocardial infarction diagnosed during the first physician encounter or during subsequent encounters, and acute myocardial infarction diagnosed only as a comorbidity. We also calculated what proportion of deaths from acute myocardial infarction occurred in people who had been in hospital on or within the 28 days preceding death, and whether acute myocardial infarction was one of the recorded diagnoses in such admissions.

**Findings:**

Acute myocardial infarction was diagnosed in the first physician encounter in 307 496 (69%) of 446 744 admissions with a diagnosis of acute myocardial infarction, in the second or later physician encounter in 52 374 (12%) admissions, and recorded only as a comorbidity in 86 874 (19%) admissions. Patients with comorbid diagnoses of acute myocardial infarction had two to three times the case-fatality rate of patients in whom acute myocardial infarction was a primary diagnosis. 135 950 deaths were recorded as being caused by acute myocardial infarction as the underlying cause of death, of which 66 490 (49%) occurred in patients who were in hospital on the day of death or in the 28 days preceding death. AMI was the primary diagnosis in 32 695 (49%) of these 66 490 patients (27 678 [42%] diagnosed in the first physician encounter and 5017 [8%] in a second or subsequent encounter), was a comorbid diagnosis in 12 118 (18%), and was not mentioned at all in the remaining 21 677 (33%). The most common causes of admission in people who did not have an acute myocardial infarction diagnosis but went on to die of acute myocardial infarction as the underlying cause of death were other circulatory conditions (7566 [35%] of 21 677 deaths), symptomatic diagnoses including non-specific chest pain, dyspnoea and syncope (1368 [6%] deaths), and respiratory disorders (2662 [12%] deaths), mainly pneumonia and chronic obstructive airways disease.

**Interpretation:**

As many acute myocardial infarction deaths occurring within 28 days of being in hospital follow a non-acute myocardial infarction admission as follow an acute myocardial infarction admission. These people are often diagnosed with other circulatory disorders or symptoms of circulatory disturbance. Further investigation is needed to establish whether there are symptoms and information that can be used to predict the risk of a fatal acute myocardial infarction in such patients, which can contribute to reducing the mortality burden of acute myocardial infarction.

**Funding:**

Wellcome Trust, Medical Research Council, Public Health England, National Institute for Health Research.

## Introduction

Acute myocardial infarction is a leading cause of hospital admissions and mortality in the UK and around the world.^1^ Multimorbidity is common in patients with a primary diagnosis of acute myocardial infarction and affects both treatment and prognosis.^2^ Furthermore, acute myocardial infarction itself might occur as comorbidity or subsequent to admission for other disorders such as pneumonia, renal failure, or hip fracture.

Rapid diagnosis, treatment, and early revascularisation can substantially improve the survival of patients with acute myocardial infarction.^3^, ^4^, ^5^ Evidence is also emerging that patients with acute myocardial infarction events that occur during hospital admissions for other disorders might receive less streamlined care and have worse mortality outcomes^6^, ^7^ than do those admitted with a primary diagnoses of acute myocardial infarction. There is, however, little information available on how long after admission acute myocardial infarction events are diagnosed,^8^ how many are only comorbidities to other primary diagnosis, and how case fatality varies based on whether acute myocardial infarction is a primary or comorbid diagnosis and when it is diagnosed. Information is also scarce on how much of the mortality burden of acute myocardial infarction is among these different types of patients admitted to hospital versus those with no admission. We aimed to address these knowledge gaps using a follow-back and follow-forward national data linkage study of hospital admissions and mortality data.

**Research in context**

**Evidence before this study**

We searched the Ovid Medline database (1946 to Feb 15, 2014) using the Medical Subject Headings (MeSH) terms “acute coronary syndrome” or “coronary artery disease” or “myocardial infarction” and “mortality” or “cause of death” or “hospital mortality” or “survival rate” or “incidence” or “registries” or “medical record linkage/medical record systems, computerised/electronic health records”, with no language restrictions. We also identified reports to the UK Government, publications from the Department of Health and the Office for National Statistics, and grey literature, using searches on Google Scholar or through citations in peer-reviewed publications. We identified three types of studies. Record linkage studies in various high-income countries, including Denmark, England, New Zealand, Scotland, and Sweden, have quantified acute myocardial infarction event and death rates for entire countries. Some of these studies have also analysed the role of comorbidity; to our knowledge, these studies have not analysed the role of whether acute myocardial infarction was diagnosed immediately after admission or later. We did not find a population-based study that had quantified the share of deaths due to acute myocardial infarction that were preceded by a recent hospital encounter with no mention of acute myocardial infarction. Second, we found studies based on acute myocardial infarction registries, which typically do not include out-of-hospital deaths due to acute myocardial infarction or deaths due to acute myocardial infarction in people admitted with disorders other than acute myocardial infarction. Finally, community-based surveillance studies have used a combination of death registries, hospital records, and review of medical records to estimate acute myocardial infarction event rates and its clinical outcomes in specific subnational populations.

**Added value of this study**

We report case-fatality rates for comprehensive categories of patients in whom a diagnosis of acute myocardial infarction was recorded at any point during their stay. To our knowledge, our study is the first to report the share of deaths from acute myocardial infarction preceded by recent or current hospital admission with no mention of acute myocardial infarction.

**Implications of all the available evidence**

In most high-income countries, about half of deaths due to acute myocardial infarction occur without a recent hospital admission. Of the deaths preceded by a recent or current admission, acute myocardial infarction is not mentioned as a diagnosis in about a third of them. Case-fatality rate was significantly higher in patients with acute myocardial infarction recorded only as a comorbidity than in those for whom acute myocardial infarction was a primary diagnosis. Improvements in the care of people admitted with an acute myocardial infarction, especially as a primary diagnosis, will only be able to affect about a third of deaths.

English (as well as other UK) hospital admission data cover almost the entire population and are uniquely detailed because they sequentially record all physician encounters during the hospital admission, which allows the time between initial admission and diagnosis of acute myocardial infarction to be detected. Multiple disorders can be coded in each physician encounter, in which acute myocardial infarction might be the primary disorder or a comorbidity of another, possibly more serious or urgent, diagnosis. This level of detail contrasts with the single discharge diagnosis reported in hospital data in most countries, in which data about timing of diagnosis and comorbid disorders are lost. We used the richness of the dataset to investigate the clinical outcomes of patients who receive an early diagnosis of acute myocardial infarction as the primary cause of admission during the first physician encounter, compared with patients with a diagnosis in the second or subsequent physician encounter, or with a solely comorbid diagnosis. We also assessed what proportion of deaths due to acute myocardial infarction occurred in people who were in hospital on the day of death or during the previous 28 days, and the diagnoses recorded in such patients.

## Methods

### Data sources

We used national data on hospital admissions and mortality in England linked at the individual level. We obtained data for hospital episode statistics from the UK Small Area Health Statistics Unit (SAHSU), supplied by the Health and Social Care Information Centre (HSCIC; now NHS Digital). Hospital episode statistics data provide information on all admissions to the UK National Health Service (NHS), a publicly funded health-care system that serves all residents of the country.

The basic unit of data in hospital episode statistics from England is the finished consultant episode. A new finished consultant episode is recorded every time a patient moves from the care of one physician to another, irrespective of whether the move is within the same hospital or involves transfer to another hospital. For example, a patient admitted under the care of an acute physician and then transferred to the care of a cardiologist will have two finished consultant episodes. If the patient is then sent to a tertiary hospital for angiography, a third finished consultant episode will be recorded along with a new admission. For each finished consultant episode, a primary diagnosis for the physician encounter is recorded with use of the International Classification of Diseases 10th revision (ICD10) codes (ICD10 code 1), as well as up to 19 secondary diagnoses (ICD10 code 2–20; appendix p 3). If the same attending physician makes a new diagnosis, this either replaces the physician's existing primary diagnosis or is added as a comorbid diagnosis in the same finished consultant episode.

We used a standard grouping algorithm^9^, ^10^, ^11^ to collapse finished consultant episodes into continuous spells of care, such that each admitted person was counted only once irrespective of how many times they were transferred between physicians and hospitals (appendix p 3). The duration of an acute myocardial infarction event is defined as 28 days in ICD10. Thus, if two acute myocardial infarction admissions for the same individual occurred within 28 days of each other, these admissions were counted as part of the same acute episode and the timing for the most recent admission was used to calculate case-fatality rate. We used the timing for the most recent admission to avoid associating two fatal events with a single patient. We use the term hospital admission to refer to a continuous spell of care. Hospital episode statistics data are coded in accordance with national coding rules, with codes based on ICD 10.^12^ On the basis of results from previous validation studies,^13^, ^14^ we used the ICD10 codes I21 and I22 to identify acute myocardial infarction events.

We also used mortality data from SAHSU, supplied by the Office for National Statistics (ONS). These data include all deaths in England. We used the underlying cause for each death record and identified deaths attributed to acute myocardial infarction using the same ICD codes as for acute myocardial infarction diagnoses. England has one of the most rigorous and systematic vital registration systems worldwide, with clear algorithms to assign underlying cause of death from the multiple causes mentioned on the death certificate. Estimates suggest that only 10–15% of assigned underlying causes in data from England are not true pathophysiological causes of death.^15^ In particular, organ failure is rarely used as an underlying cause of death in mortality statistics from England (0·4% of all deaths in 2010).

**Figure 1:**
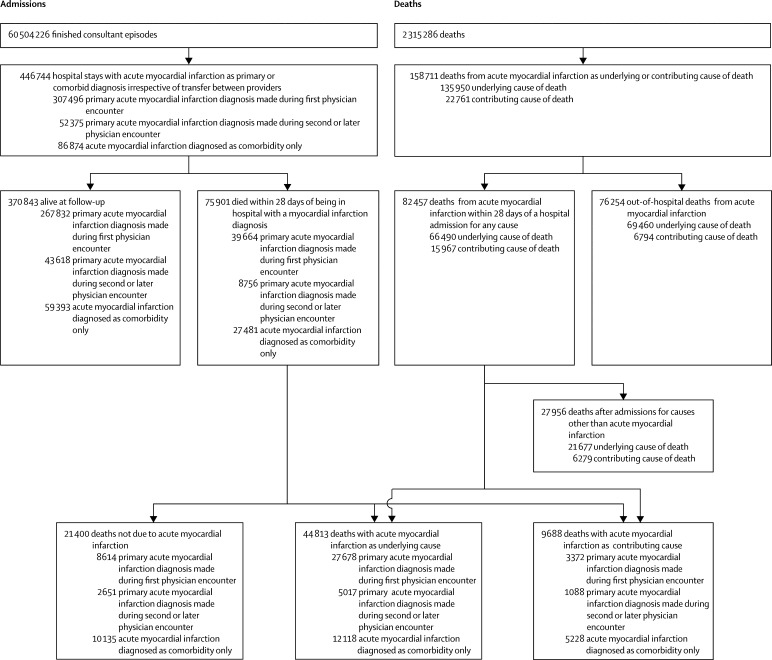
Schematic of hospital admissions and deaths Data only include people aged 35 years and older. Deaths and admissions were from Jan 1, 2006, to Dec 31, 2010. Admissions linked to death from acute myocardial infarction within this period might have occurred up to 28 days before the start of this period and were included. Deaths linked to admissions within this period might have occurred up to 28 days after the end of the period and were included. We used a continuous inpatient spell algorithm to collapse finished consultant episodes into admissions and then collapsed them further so that admissions within 28 days of the index event were counted as part of the same event. A breakdown of the numbers in each category is available in the appendix (p 2).

The final dataset consisted of all hospital admissions with acute myocardial infarction and all deaths from acute myocardial infarction from 2006 to 2010 in people aged 35 years and older (figure 1). Record linkage between hospital admission and deaths was provided by the HSCIC and included deaths registered up to March 31, 2012. In England, late registration of death can occur if there is an inquest into the cause of death. Out-of-hospital deaths due to acute myocardial infarction were classified as those without any hospital admissions in the preceding 28 days. Not only is 28 days consistent with the definition of acute myocardial infarction used in the ICD, it is also the duration used to define an acute event in the WHO Multinational MONItoring of trends and determinants in CArdiovascular disease (MONICA) Project.

Data use was approved by the National Research Ethics Service (reference 12/LO/0566 and 12/LO/0567) and the Health Research Authority Confidentially Advisory Group (HRA-CAG) for Section 251 support (HRA-14/CAG/1039).

### Statistical analysis

We assessed the proportion of deaths due to acute myocardial infarction that occurred in people who were in a hospital in the previous 28 days, including on the day of death, and whether acute myocardial infarction was one of the recorded diagnoses in such patients. For those deaths without an acute myocardial infarction diagnosis, we assessed the diagnoses recorded in the final physician encounter.

We also calculated event rates per 100 000 population and case-fatality rate by age group and sex for primary acute myocardial infarction diagnosed during the first physician encounter, primary acute myocardial infarction diagnosed during subsequent encounters, and acute myocardial infarction diagnosed only as a comorbidity. We calculated the rates of hospital admissions for primary and comorbid acute myocardial infarction using mid-year age-sex-specific population estimates provided by the ONS. We calculated case-fatality rate as the proportion of admissions in each group that resulted in death within 28 days. We estimated 95% CIs with the assumption that the observed number of events or deaths followed a Poisson distribution.

In a sensitivity analysis, we excluded those aged 85 years and older from the sample to investigate whether our findings were largely driven by deaths in the very elderly. We also examined the effects of restricting admissions to those marked as emergency and to those with duration greater than 1 day because suggestions have previously been made that short and non-emergency admissions might include patients who are investigated for acute myocardial infarction but do not actually have it.^16^, ^17^, ^18^

### Role of the funding source

The funder of the study had no role in study design, data collection, data analysis, data interpretation, or writing of the report. PA and MD had full access to all the data in the study and ME had final responsibility for the decision to submit for publication.

## Results

**Figure 2:**
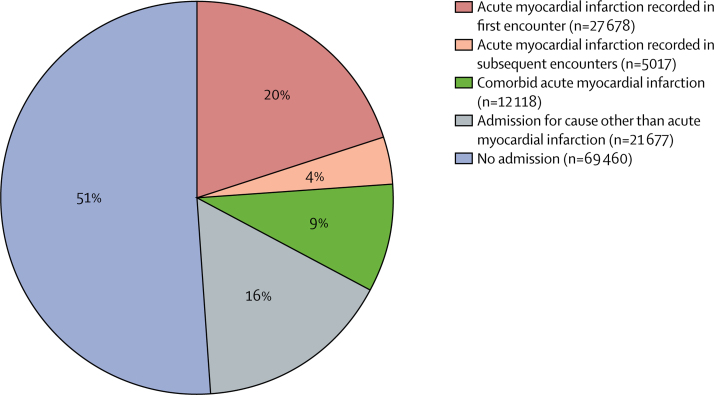
Hospital admissions in the 28 days preceding death from acute myocardial infarction Includes 135 950 deaths with acute myocardial infarction as the underlying cause in people aged 35 years and older.

**Figure 3:**
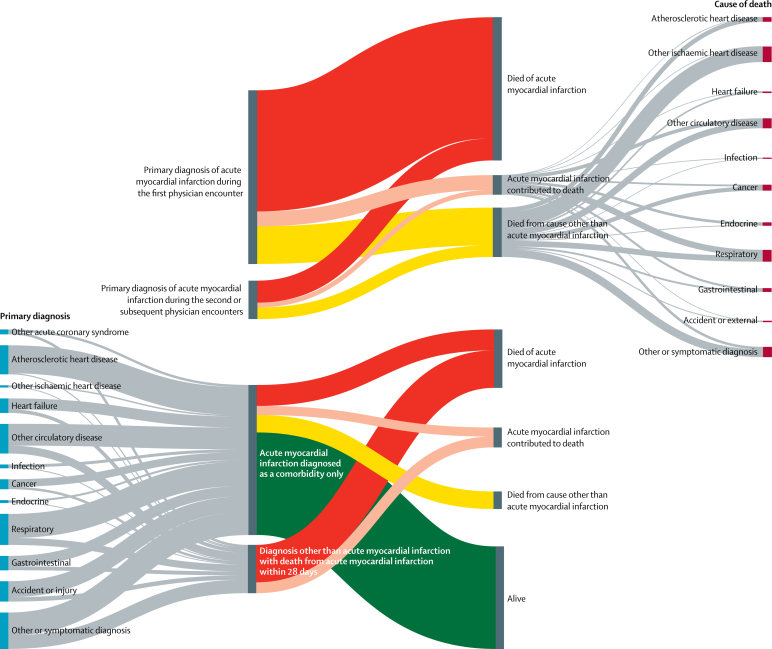
Diagnostic flow of patients admitted to hospital with acute myocardial infarction and those dying of acute myocardial infarction The top section shows underlying causes of death for patients with a primary acute myocardial infarction diagnosis recorded during the first physician encounter and during subsequent encounters who died within 28 days. The proportion of patients with a primary acute myocardial infraction diagnosis who remained alive are not shown here, as their number is large, but are given in the table and the appendix (p 2). The bottom section shows primary diagnoses for patients with acute myocardial infarction diagnosed as a comorbidity and for patients admitted with a cause other than acute myocardial infarction admission who died from acute myocardial infarction within 28 days. Grey bands are proportionate to the number of patients in each category. The vertical bars on the far left show the specific primary hospital diagnoses, and those on the far right show the specific causes of death for people whose primary diagnosis or cause of death was not acute myocardial infarction.

Acute myocardial infarction was recorded as the underlying cause of 135 950 deaths during the analysis (figure 1, table). Of the people who died from acute myocardial infarction, 69 460 (51%) died without a hospital admission in the preceding 28 days and 66 490 (49%) died within 28 days of having been in hospital (figure 2). 59 187 (89%) of the deaths within 28 days of having been in a hospital occurred while the patient was still in the hospital. The hospital records for the 66 490 patients who were in hospital in the 28 days before death from acute myocardial infarction showed that acute myocardial infarction was the primary diagnosis in 32 695 (49%) of these deaths (27 678 diagnosed in the first physician encounter and 5017 in a second or subsequent encounter), a comorbid diagnosis in 12 118 (18%), and not mentioned at all in the remaining 21 677 (33%). 16 530 (76%) of the 21 677 patients without a mention of acute myocardial infarction in their previous records were still in hospital at the time of death. The most common diagnoses in people without a diagnosis of acute myocardial infarction who went on to have acute myocardial infarction as the underlying cause of death were other circulatory disorders (7566 [35%] of 21 667 patients), including heart failure and atrial fibrillation; symptomatic diagnoses (6434 [30%] patients), 1368 of which were non-specific chest pain, dyspnoea, syncope, and haemodynamic disturbance or abnormal heart sounds; respiratory disorders (2662 [12%] patients), mostly consisting of pneumonia and chronic obstructive airways disease; and injuries (1912 [9%] patients), especially hip fractures (figure 3).

**Table:** Acute myocardial infarction events by event type, age, and sex

		**Primary diagnosis of acute myocardial infarction**						**Acute myocardial infarction diagnosed as comorbidity only**			**Diagnosis other than acute myocardial infarction with death from acute myocardial infarction within 28 days**	**Acute myocardial infarction death with no previous admission**
		Diagnosed at first encounter			Diagnosed at subsequent encounters			Non-fatal	Acute myocardial infarction death	Death from other cause		
		Non-fatal	Acute myocardial infarction death	Death from other cause	Non-fatal	Acute myocardial infarction death	Death from other cause					

Men												
	35–44 years	9179	104	56	949	12	6	1230	30	34	104	1093
	45–54 years	26 619	509	149	2879	30	33	3587	150	147	392	3435
	55–64 years	41 545	1456	521	4613	141	114	6189	504	571	1163	7159
	65–74 years	42 004	2856	1277	6163	439	367	8136	1175	1632	2679	10 119
	75–84 years	37 709	5449	2572	7089	975	781	9676	2489	3246	4468	13 610
	≥85 years	16 083	4017	1952	3349	835	613	4797	1826	2286	3101	7379
	Total ≥35 years	173 139	14 391	6527	25 042	2432	1914	33 615	6174	7916	11 907	42 795
Women												
	35–44 years	1917	46	13	267	4	4	317	12	28	51	229
	45–54 years	6101	147	67	869	22	14	1003	61	98	135	601
	55–64 years	12 178	488	182	1733	62	67	2257	188	323	452	1528
	65–74 years	20 320	1571	689	3581	271	228	4678	653	983	1273	3988
	75–84 years	30 559	4662	1984	6525	902	650	8961	2119	2667	3477	9529
	≥85 years	23 618	6373	2524	5601	1324	862	8562	2911	3348	4382	10 790
	Total ≥35 years	94 693	13 287	5459	18 576	2585	1825	25 778	5944	7447	9770	26 665
Total		267 832	27 678	11 986	43 618	5017	3739	59 393	12 118	15 363	21 677	69 460

855 523 (1%) of 60 504 226 hospital episode statistics records mentioned acute myocardial infarction as either a primary or comorbid diagnosis (figure 1). After accounting for transfer between physicians and providers, we collapsed these records into 446 744 continuous spells of care in which acute myocardial infarction was recorded as a primary or comorbid diagnosis. The patient died in 75 901 (17%) of these admissions. In 359 870 (81%) of 446 744 acute myocardial infarction admissions, the primary diagnosis was acute myocardial infarction. Of these 359 870 patients with primary diagnoses of acute myocardial infarction, the diagnosis was made at the first physician encounter for 307 496 (85%) patients (69% of all 446 744 admissions for acute myocardial infarction) and in the second or subsequent encounter for the other 52 375 (15%) patients (12% of all 446 744 admissions for acute myocardial infarction). Diagnoses of acute myocardial infarction that were made in the second or subsequent physician encounter were more common in elderly people than in young and middle-aged people, and represented a greater proportion of all acute myocardial infarction diagnoses in women than in men (table).

Comorbid diagnoses of acute myocardial infarction represented 86 874 (19%) of 446 744 hospital admissions for acute myocardial infarction (47 705 [18%] of 271 150 admissions of men and 39 169 [22%] of 175 594 admissions of women). The most frequent primary causes for admission in these patients were atherosclerotic heart disease, hip fracture, pneumonia, chronic obstructive airways disease, heart failure, and stroke (figure 3). Overall, the primary causes in 37 234 (43%) of these admissions were other circulatory conditions and in the remaining 49 640 (57%) admissions they were non-circulatory causes. 23 730 (26%) of 90 881 admissions for acute myocardial infarction in patients aged 85 years or older were comorbid diagnoses, compared with 1651 (12%) of 14 208 admissions in patients aged 35–44 years.

**Figure 4:**
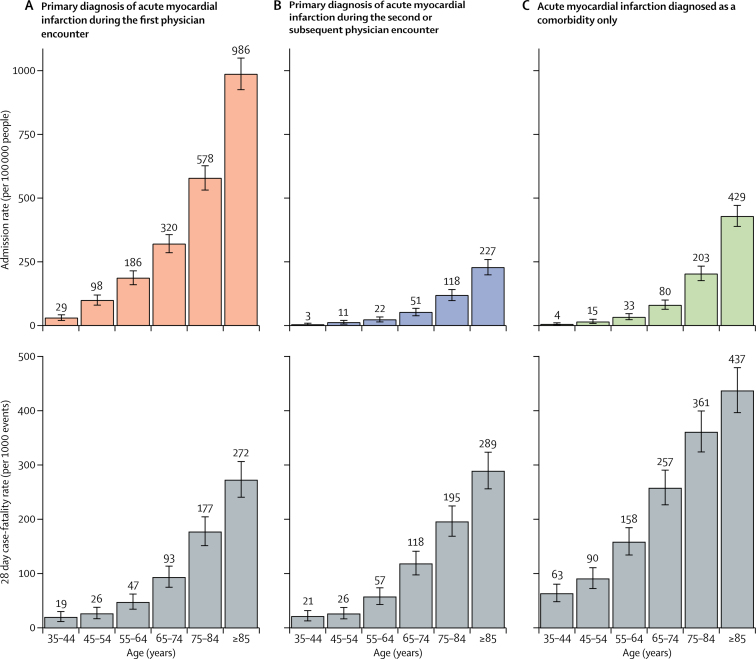
Age-specific event and 28-day case-fatality rates by age group Rates are for patients admitted to hospital with a primary diagnosis of acute myocardial infarction recorded during the first physician encounter or during subsequent physician encounters and patients for whom acute myocardial infarction was diagnosed only as a comorbidity. Results by sex are available in the appendix (p 1).

Admission and case-fatality rates for hospitalised patients are shown in figure 4. The 28 day case-fatality rate for death from any cause in patients with a primary diagnosis of acute myocardial infarction during the first physician encounter ranged from 19 deaths per 1000 events in 35–44 year olds to 272 deaths per 1000 events in patients aged 85 years and older (figure 4). Case-fatality rates were slightly higher in patients with a primary diagnosis of acute myocardial infarction made in the second or subsequent physician encounter than in those with a diagnosis made at the first encounter (figure 4).^8^ By contrast, case-fatality rates were substantially higher in patients with acute myocardial infarction recorded only as a comorbidity than in patients with primary acute myocardial infarction diagnosis made in the first physician encounter, ranging from 63 deaths per 1000 events in patients aged 35–44 years to 437 deaths per 1000 events in those aged 85 years or older. In people younger than 65 years, case-fatality rates were higher in women than in men, especially for comorbid acute myocardial infarction (appendix p 1). Case-fatality rates for the men and women equalised as age increased.

11 265 (23%) of 48 420 deaths within 28 days of being in hospital for acute myocardial infarction were certified to causes other than acute myocardial infarction, frequently chronic ischaemic heart diseases, other circulatory conditions, atherosclerotic heart disease, respiratory disorders, and cancer, whereas acute myocardial infarction was a contributing cause in 4460 (9%) of 48 420 deaths (figure 3).

**Figure 5:**
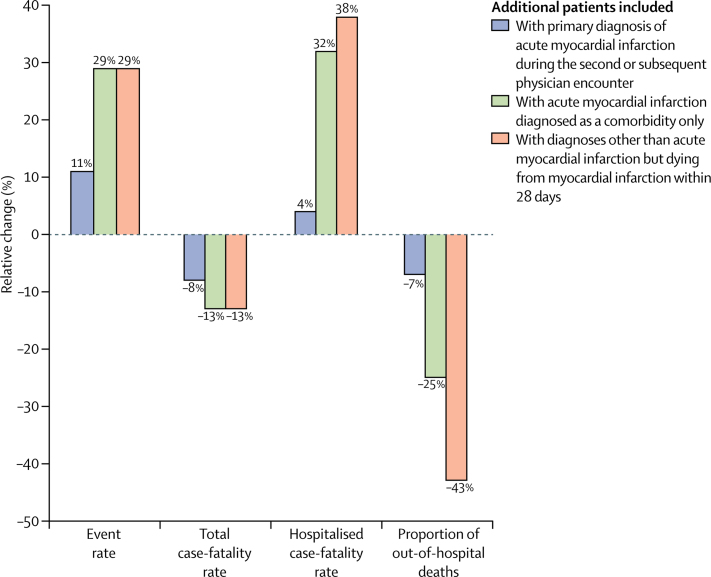
Effects of including different patient groups on national estimates of epidemiological parameters for acute myocardial infarction Bars show the relative change in national acute myocardial infarction event rate, total case-fatality rate, hospitalised case-fatality rate, and proportion of out-of-hospital deaths with incremental inclusion of additional types of patients. All comparisons are relative to patients with a primary diagnosis of acute myocardial infarction recorded during the first physician encounter.

The crude acute myocardial infarction event rate in people aged 35 years or older with a primary diagnosis of acute myocardial infarction in the first physician encounter, including fatal and non-fatal acute myocardial infarction admissions and out-of-hospital deaths from acute myocardial infarction, was 288 cases (95% CI 287–289) per 100 000 people; the crude case-fatality rate was 356 deaths (354–358) per 1000 events. Figure 5 shows the effects of including diagnoses of acute myocardial infarction made in second or subsequent physician encounters and comorbid diagnoses on the national estimates of acute myocardial infarction event rate, case-fatality rate, hospitalised case fatality rate, and proportion of out-of-hospital deaths.

Our sensitivity analysis excluding patients aged 85 years and older resulted in a higher proportion of acute myocardial infarction deaths not preceded by any admission (51 291 [55%] of 93 012 deaths) than in our main analyses (69 460 [51%] of 135 950 deaths). However, the proportion of deaths occurring after an admission for a cause other than acute myocardial infarction admission or after a comorbid acute myocardial infarction admission (21 575 [23%] of 93 012 deaths) remained roughly equal to the proportion occurring after an admission for acute myocardial infarction (20 146 [22%] of 93 012 deaths; appendix p 4).

In our sensitivity analysis excluding short stay (less than 1 day) admissions, the admission rate for acute myocardial infarction diagnosed as a primary disorder was 242 cases per 100 000 people, which was 3% lower than in our main analysis (249 cases per 100 000 people). When we excluded non-emergency admissions, the admission rate was 236 cases per 100 000 people, which was a 5% reduction. These sensitivity analyses had little effect on case-fatality rates (data not shown). It is now feasible to rapidly diagnose, treat, and safely discharge a low-risk patient who has had a true acute event, within 24–48 h,^19^, ^20^, ^21^ and as such, exclusion of short-stay and non-emergency patients could lead an underestimation of acute myocardial infarction event rates.

## Discussion

The results of our national data-linkage study show the clinical trajectory of the different presentations of acute myocardial infarction in England. 49% of all people with fatal acute myocardial infarction were in hospital at the time of their death or in the 28 days preceding it and the other 51% of people who died of acute myocardial infarction deaths did not have a recent admission. Acute myocardial infarction was not mentioned as a diagnosis in 33% of deaths with a recent admission (ie, 16% of all acute myocardial infarction deaths). In about 12% of all patients admitted to hospital with acute myocardial infarction, acute myocardial infarction was diagnosed in the second or later physician encounter; it was recorded only as a comorbidity in a further 19%.

Our results are based on a large dataset that covers almost the entire population of England, where admissions to private hospitals represent only a very small proportion of total admissions, especially for acute disorders such as acute myocardial infarction. Population-based record linkage can simultaneously capture non-fatal acute myocardial infarctions, and both in and out of hospital fatal ones, allowing the clinical trajectory of the disorder to be fully followed up. Studies in a few countries where record linkage is feasible have produced estimates of acute myocardial infarction event rates, case-fatality rates, and proportion of out-of-hospital deaths,^17^, ^22^, ^23^, ^24^, ^25^, ^26^, ^27^ as well as other measures of quality of care.^21^, ^28^, ^29^ Our data have the additional advantage of allowing us to analyse the role of acute myocardial infarction that is diagnosed in the second or later physician encounter or only as a comorbidity, which have been excluded from previous analyses in England.

The use of routine data, although necessary for national surveillance, also leads to some limitations. Clinical criteria defined for the case-ascertainment of acute myocardial infarction in cohort studies do not translate well to routine ICD10 codes. Furthermore, ICD codes provide a standardised summary of a diagnosis but do not specify the underlying investigations and laboratory results—eg, ST elevation acute myocardial infarction cannot be distinguished from non-ST elevation acute myocardial infarction in ICD coding. We were thus unable to verify acute myocardial infarction events with electrocardiogram and laboratory findings, and we were reliant on the routine coding of hospital episode statistics and mortality data. The quality of hospital data varies by disorder, with common diagnoses such as acute myocardial infarction being better coded than rarer ones.^30^, ^31^ Results from linkage studies in Scotland and Wales, which used data similar to ours, show high accuracy for the diagnosis of acute myocardial infarction.^32^, ^33^ Furthermore, in two systematic reviews that examined studies comparing disease registers to routinely coded acute myocardial infarction, sensitivity and positive predictive values were 79–95%, with hospital data seeming to have better validity than death records.^13^, ^14^ For deaths, it is possible that certifying doctors use acute myocardial infarction as a convenient catch-all terminal cause when they have insufficient information about the deceased's recent medical history. Evidence suggests that acute myocardial infarction might be over-assigned as a cause of death in some subgroups and under-assigned in others.^14^, ^34^ Guided by our results on subgroups with admissions for causes other than acute myocardial infarction preceding death from acute myocardial infarction, in-depth studies are needed to verify whether these admissions represent missed opportunities for the diagnosis and treatment of acute myocardial infarction versus misclassification of cause of death. Recorded cases aside, some studies have estimated that routinely collected hospital and mortality data might underestimate the overall incidence of acute myocardial infarction by 10–23%.^35^, ^36^ Finally, linkage itself is an imperfect process and record linkage in England fails to match 2·3% of deaths to their preceding admission.^37^

Our findings have three main implications. First, our results emphasise the under-investigated role of fatal acute myocardial infarction events with a recent or even current hospital admission with no mention of acute myocardial infarction or a mention only as a comorbidity. These patients make up an equal share to the mortality burden of acute myocardial infarction within 28 days of an admission with a diagnosis of acute myocardial infarction. Their hospital records often contain primary diagnoses of circulatory disorders that share risk factors with acute myocardial infarction, or mention non-specific chest pain, dyspnoea, or syncope, which might herald the impending death from acute myocardial infarction.^38^ The case-fatality rate for acute myocardial infarction diagnosed as a primary disorder is already relatively low, so further improvements in hospital management might only produce small reductions in mortality burden. Hospital protocols aimed at the management of acute myocardial infarction might not benefit the clinical trajectory of patients with circulatory disorders or haemodynamic instability for whom no acute myocardial infarction was diagnosed during admission. If the available clinical information can be used to prudently generate a high clinical index of suspicion, followed by risk stratification and early management, these admissions could represent opportunities to reduce the mortality burden of acute myocardial infarction.

Second, about 51% of all mortality from acute myocardial infarction is attributable to out-of-hospital deaths without a recent hospital admission. The length of delay between symptom onset and a call for help has changed little since the 1980s.^39^, ^40^, ^41^ More than half of these patients have symptoms (commonly chest pain, breathlessness, or a general feeling of unwellness) for a duration of more than 15 min before collapse.^42^ Strategies to shorten pre-hospital delays could result in substantial improvements in acute myocardial infarction survival in this group.^43^

Third, patients with comorbid diagnoses of acute myocardial infarction constitute roughly 19% of all admissions related to acute myocardial infarction and have case-fatality rates two to three times higher than those of patients with a main diagnosis of acute myocardial infarction. Patients with comorbid acute myocardial infarction diagnoses tend to be older and are more likely to be women than patients with main diagnoses and often have a stressor condition such as hip fracture or pneumonia recorded as the primary reason for admission. The universal definition of myocardial infarction subclassifies acute myocardial infarction into type 1 acute myocardial infarction, in which cardiac ischaemia is caused by the formation of an acute thrombus in the coronary arteries, and type 2, in which it is caused by an imbalance in the supply and demand for myocardial oxygen.^44^ Although we cannot be certain without full record review, the timing and secondary prioritisation of most acute myocardial infarctions recorded as comorbid events would suggest that they belong to the type 2 category, especially those accompanying disorders such as atrial fibrillation. The optimal management of type 2 acute myocardial infarction is currently uncertain, with risk assessment and optimisation of haemodynamic status remaining the mainstay of treatment for these patients.

The underlying method to reduce mortality after acute myocardial infarction is timely contact with the health system and diagnosis of the acute myocardial infarction. Our findings on the distributions of deaths from acute myocardial infarction show that substantial reductions in acute myocardial infarction mortality will require attention to the large proportion of these deaths that are not preceded by a hospital admission or are preceded by an admission for a another cause.
