# Supplementary material for: Acute myocardial infarction hospital admissions and deaths in England: a national follow-back and follow-forward record-linkage study
Source: Lancet Public Health. 2017 Mar 1;2(4):e191–201. doi: 10.1016/S2468-2667(17)30032-4 (PMC6196770; doi:10.1016/S2468-2667(17)30032-4)

Appendix Table 1: Event rates and case-fatality for subtypes of acute myocardial infarction (AMI) admission by age and sex.

Age group	Admitted with main diagnosis of AMI				Admitted with comorbid AMI diagnosis		Admitted with diagnosis other than AMI and died of AMI within 28 days
	Diagnosed first encounter		Diagnosed subsequent encounters				
	Event rate (95% CI) per 100,000	Case-fatality (95% CI) (%)	Event rate (95% CI) per 100,000	Case-fatality (95% CI) (%)	Event rate (95% CI) per 100,000	Case-fatality (95% CI) (%)	Event rate (95% CI) per 100,000
				**Men**			
35-44	49 (48-50)	1.7 (1.5-2)	5 (5-5)	1.9 (1.1-2.9)	7 (6-7)	4.9 (3.8-6.3)	1 (0-1)
45-54	161 (159-163)	2.4 (2.2-2.6)	17 (17-18)	2.1 (1.6-2.7)	23 (22-24)	7.6 (6.8-8.6)	2 (2-3)
55-64	291 (288-294)	4.5 (4.3-4.7)	33 (32-33)	5.2 (4.6-5.9)	49 (47-50)	14.8 (13.9-15.7)	8 (7-8)
65-74	449 (445-453)	9.0 (8.7-9.2)	68 (66-69)	11.6 (10.8-12.4)	107 (105-109)	25.7 (24.7-26.6)	26 (25-27)
75-84	750 (743-757)	17.5 (17.2-17.9)	145 (142-148)	19.9 (18.9-20.8)	253 (249-257)	37.2 (36.3-38.2)	73 (71-75)
85+	1,276 (1,259-1,293)	27.1 (26.4-27.8)	278 (270-286)	30.2 (28.7-31.8)	515 (505-526)	46.2 (44.8-47.6)	179 (173-186)
				**Women**			
35-44	10 (10-11)	3.0 (2.3-3.9)	1 (1-2)	2.9 (1.3-5.7)	2 (2-2)	11.2 (8.0-15.3)	0 (0-0)
45-54	37 (36-38)	3.4 (2.9-3.9)	5 (5-6)	4.0 (2.8-5.5)	7 (6-7)	13.7 (11.6-16)	1 (1-1)
55-64	83 (82-85)	5.2 (4.8-5.6)	12 (12-13)	6.9 (5.8-8.2)	18 (17-19)	18.5 (16.9-20.1)	3 (3-3)
65-74	201 (198-204)	10.0 (9.6-10.4)	36 (35-37)	12.2 (11.2-13.4)	56 (55-58)	25.9 (24.7-27.2)	11 (11-12)
75-84	451 (446-455)	17.9 (17.4-18.3)	98 (96-100)	19.2 (18.3-20.2)	167 (164-169)	34.8 (33.8-35.8)	42 (41-44)
85+	854 (845-863)	27.4 (26.8-27.9)	205 (200-209)	28.1 (26.9-29.3)	389 (383-396)	42.2 (41.2-43.3)	115 (112-119)

Appendix Table 2: Breakdown of acute myocardial infarction (AMI) admissions and deaths included in the analysis.

	Primary AMI admission		Comorbid AMI admission	Admitted with diagnosis other than AMI and died of AMI within 28 days	No preceding admission in last 28 days	**Total**
	Diagnosed first encounter	Diagnosed subsequent encounters				
Died: AMI as underlying cause	27,678	5,017	12,118	21,677	69,460	**135,950**
Died: AMI as contributing cause	3,372	1,088	5,228	6,279	6,794	**22,761**
Died: Non-AMI cause	8,614	2,651	10,135	0	NA	
Alive at 28 days	267,832	43,618	59,393	0	NA	
**Total**	**307,496**	**52,374**	**86,874**	**27,956**	**76,254**	**158,711**

Appendix Figure 1: Example of the structure of the English Hospital Episode Statistics (HES) data, showing the multiple finished consultant episodes (FCE) and diagnostic code positions available for analysis. The arrows indicate how continuous spells of care, which define a complete admission for each patient, were constructed. AMI = acute myocardial infarction, ICD = international classification of diseases, MI = myocardial infarction, NOF = neck of femur, PVD = peripheral vascular disease.

Primary diagnosis (ICD10 code 1); Secondary (co-morbid) diagnoses (ICD10 code 2 to ICD10 code 20)

Finished consultant episodes ↓

Patient ID	Hospital ID	Admission Number	Admission date	Discharge date	Episodes per admission	Consultant's speciality	ICD10 code 1	ICD10 code 2	ICD10 code 3	ICD10 code ...	ICD10 code 20	Continuous spell of care
Patient A	X	1	3-Feb-10	5-Feb-10	1	Acute medicine	AMI	PVD	Diabetes	-	-	1
Patient A	X		3-Feb-10	5-Feb-10	2	Cardiology	AMI	PVD	Diabetes	-	-	
Patient A	Y	2	5-Feb-10	7-Feb-10	1	Interventional cardiology	Athersclerotic heart disease	Anterior MI	PVD	Diabetes	-	
Patient B	Z	3	8-Feb-10	3-Mar-10	1	General Surgery	Cholecystitis	Biliary Obstruction	AMI	-	-	2
Patient B	Z		8-Feb-10	3-Mar-10	2	Gastroenterology	Cholecystitis	Acute MI	-	-	-	
Patient B	Z		8-Feb-10	3-Mar-10	3	Care of the Elderly	Delirium	Cholecystitis	AMI	-	-	
Patient B	Z		8-Feb-10	3-Mar-10	4	Orthopaedics	Fractured NOF	Delirium	Cholecystitis	AMI	-	
Patient B	Z		8-Feb-10	3-Mar-10	5	Rehabilitation	Fractured NOF	Delirium	Cholecystitis	AMI	-	

Diagnostic positions →

Appendix Figure 2: Distribution of hospital admissions in the 28 days preceding death with acute myocardial infarction (AMI)as underlying cause (93,012 deaths) restricted todeaths in people aged 35-84 years.

-

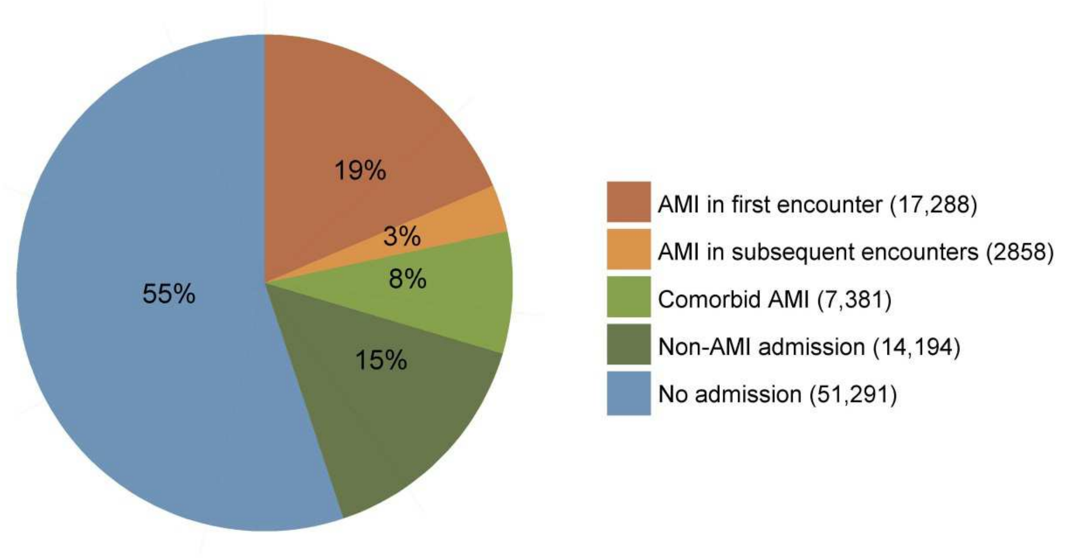

Supplement: Supplementary appendix [file mmc1.pdf]
